# TRRUST: a reference database of human transcriptional regulatory interactions

**DOI:** 10.1038/srep11432

**Published:** 2015-06-12

**Authors:** Heonjong Han, Hongseok Shim, Donghyun Shin, Jung Eun Shim, Yunhee Ko, Junha Shin, Hanhae Kim, Ara Cho, Eiru Kim, Tak Lee, Hyojin Kim, Kyungsoo Kim, Sunmo Yang, Dasom Bae, Ayoung Yun, Sunphil Kim, Chan Yeong Kim, Hyeon Jin Cho, Byunghee Kang, Susie Shin, Insuk Lee

**Affiliations:** 1 Department of Biotechnology, College of Life Science and Biotechnology, Yonsei University, Seoul, Korea

## Abstract

The reconstruction of transcriptional regulatory networks (TRNs) is a long-standing challenge in human genetics. Numerous computational methods have been developed to infer regulatory interactions between human transcriptional factors (TFs) and target genes from high-throughput data, and their performance evaluation requires gold-standard interactions. Here we present a database of literature-curated human TF-target interactions, TRRUST (transcriptional regulatory relationships unravelled by sentence-based text-mining, http://www.grnpedia.org/trrust), which currently contains 8,015 interactions between 748 TF genes and 1,975 non-TF genes. A sentence-based text-mining approach was employed for efficient manual curation of regulatory interactions from approximately 20 million Medline abstracts. To the best of our knowledge, TRRUST is the largest publicly available database of literature-curated human TF-target interactions to date. TRRUST also has several useful features: i) information about the mode-of-regulation; ii) tests for target modularity of a query TF; iii) tests for TF cooperativity of a query target; iv) inferences about cooperating TFs of a query TF; and v) prioritizing associated pathways and diseases with a query TF. We observed high enrichment of TF-target pairs in TRRUST for top-scored interactions inferred from high-throughput data, which suggests that TRRUST provides a reliable benchmark for the computational reconstruction of human TRNs.

Transcription factors (TFs) are major molecules that control the transcriptional activity of genes. The human genome is estimated to encode approximately 2,000 TFs^1^, which operate programs that change cellular states by binding to proxy or distal cis-regulatory elements (CREs) for a set of target genes. The reverse engineering of transcriptional regulatory networks (TRNs) by inferring interactions between TFs and target genes has been a key challenge in understanding the genetic regulation of complex human phenotypes. However, genome-scale regulatory circuit models remain inadequate due to the intrinsic complexities of human transcriptional regulatory programs as well as technical limitations in mapping regulatory interactions.

Interactions between TFs and CREs of target genes are generally detected by DNA-binding experiments such as chromatin immunoprecipitation (ChIP), which is often followed by microarray analysis (ChIP-chip) or deep-sequencing analysis (ChIP-seq). TF-target regulatory interactions also can be inferred from high-throughput gene expression data using a wide variety of computational algorithms^2^. Expression data have advantages over DNA-binding data in the coverage of diverse cellular contexts, which reveal disparate sets of regulatory interactions. Thus, by integrating TF-target interactions inferred from a wide variety of cellular contexts, we may effectively reconstruct genome-scale TRNs. The integrative approach of TRN modelling requires gold-standard TF-target interactions to benchmark inferred regulatory interactions from different data sets. The reconstruction of TRNs via the integration of regulatory interactions inferred from multiple data sets has previously been demonstrated in several model organisms, and databases for literature-curated TF-target interactions have played critical roles, e.g., RegulonDB^3^ for an *Escherichia coli* TRN^4^, the Yeast Proteome Database (YPD)^5^ for a *Saccharomyces cerevisiae* TRN^6^, and the Regulatory Element Database for Drosophila (REDfly)^7^ for a *Drosophila melanogaster* TRN construction^8^.

Several public databases for human TF-target interactions are currently available, including TFactS^9^, TRED^10^, HTRIdb^11^, and ORegAnno^12^. However, some of the databases include interactions inferred from high-throughput experiments, which may not be optimal for benchmarking. If we count only literature-curated interactions from those databases, then the size of the gold-standard data set may not be large enough to fairly evaluate TRNs. Therefore, we need to expand the size of our current set of gold-standard regulatory interactions for the more effective reconstruction of human TRNs, which is our main motivation for developing an extensive database of literature-curated human regulatory interactions.

TRRUST (transcriptional regulatory relationships unravelled by sentence-based text-mining) is a database of TF-target regulatory interactions identified via the manual curation of Medline abstracts. For efficient curation over a large number of Medline abstracts, we used a sentence-based text-mining approach in which text sentences that might pertain to transcriptional regulation were first extracted and then subjected to manual curation. The current version of TRRUST contains 8,015 TF-target interactions, which to our knowledge is the largest public database of literature-curated human regulatory interactions to date. Moreover, a majority of the interactions have annotations for mode-of-regulation (i.e., activation or repression). In addition, by incorporating functional or physical protein interaction networks, TRRUST performs a network analysis of TFs and their targets to provide various systemic context information for facilitating functional interpretations, including target modularity^13^, TF cooperativity^14^, and TF-pathway and TF-disease associations. Importantly, we observed that gene pairs in TRRUST are highly enriched among the top-ranked regulatory interactions inferred from high-throughput expression data, which suggests that the TRRUST data will be a useful benchmark for the computational reconstruction of human TRNs.

## Results

### TF-target interactions of the TRRUST database

**Figure 1:**
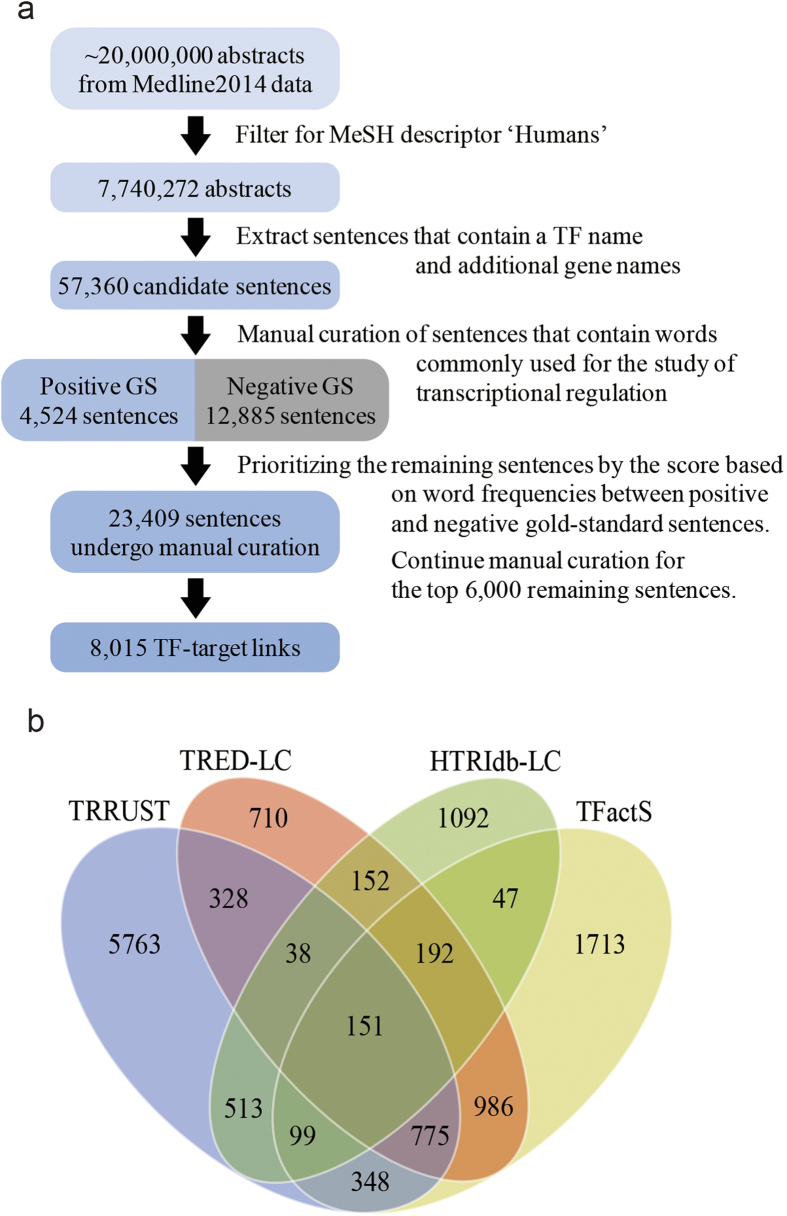
(**a**) The overall process of constructing the TRRUST database via the manual curation of Medline abstracts using a sentence-based text-mining approach is outlined. GS stands for gold-standard. (**b**) A Venn diagram illustrates the overlap of TF-target regulatory interactions from four literature-curated databases: TRRUST, TRED-LC (literature-curated interactions of TRED), HTRIdb-LC (literature-curated interactions of HTRIdb), and TFactS.

The overall process of constructing the TRRUST database is summarized in Fig. 1a. To increase the efficiency of the literature curation, we employed a ‘sentence-based text-mining’ approach, which is described in more detail in **Methods**. Briefly, we scanned ~20 million abstracts from the Medline2014 database for studies involving human biology using the MeSH descriptor ‘Humans’, which returned 7,740,270 abstracts. We then extracted 57,360 sentences that contained at least one TF name and additional gene names, which are referred to as ‘*candidate sentence*s’. The list of TF genes were derived from Ravasi *et al.*^15^, which reported manually curated TF genes from several sources: i) the TRANSFAC database; ii) genes annotated by the Gene Ontology (GO) term ‘transcription factor’; iii) genes that contain the word ‘transcription’ in the Entrez description field; and iv) manually curated TF genes by Roach *et al.*^16^ After further curation, we generated a list of 1,984 TFs for our database. False positives of the TF list would not affect the quality of our database, because TF-target interactions will be identified by manual curation.

For the given candidate sentences, we conducted a two-step text-mining procedure. In the first step, we established gold-standard candidate sentences via manual curation. This gold-standard set was updated by incoming sentences from post-manual curation, and used to prioritize incoming candidate sentences for the next round of manual curation. In the second step, we prioritized the remaining candidate sentences by a score based on the frequency difference of each word between the gold-standard positives (i.e., sentences that contain a TF and other genes for a regulatory interaction) and negatives (i.e., sentences that contain a TF and other genes but not for a regulatory interaction) (see **Methods** for details). We then continued the manual curation for an additional 6,000 candidate sentences from the top-scored sentences. In total, we identified 8,015 TF-target regulatory interactions between 748 TFs and 1,975 non-TF genes over two rounds of manual curation from 23,409 candidate sentences corresponding to 20,317 abstracts. The 6,000 sentences that were identified in the second round of manual curation can be used to update the set of gold-standard candidate sentences to further improve the retrieval rate in future manual curations by re-prioritizing sentences.

We found that TRRUST has substantially more literature-curated (LC) human TF-target regulatory interactions than other public databases: TFactS^9^, TRED^10^, HTRIdb^11^, and ORegAnno^12^ (Table 1). TRRUST contains an approximately 2.5-fold greater number of TFs and two-fold greater number of TF-target interactions than the second largest database, TFactS. We compared the data content of TRRUST with three other major public databases: TFactS, TRED, and HTRIdb (Fig. 1b). Notably, 5,763 (~72%) of the TRRUST TF-target interactions are non-overlapping with the other three databases. These results indicate that our literature curation covered a substantially larger number of Medline abstracts than these other databases.

The regulatory action of a TF either activates or represses the transcription of its target gene. Information about the mode-of-regulation may be important in interpreting the phenotype effects of TF dysregulation. Therefore, we collected mode-of-regulation information for given TF-target interactions from the abstracts, if available. Among other public TF-target databases, only TFactS includes mode-of-regulation information. Currently, 4,861 TF-target interactions in TRRUST (~60%) include mode-of-regulation annotations based on evidence from the literature: 3,180 interactions for activation, 1,881 interactions for repression, and 200 interactions for both. A TF-target link could be annotated for both activation and repression modes by independent studies, due to the differential regulatory coordination across cellular contexts.

### Target modularity and TF cooperativity in the TRRUST database

**Figure 2:**
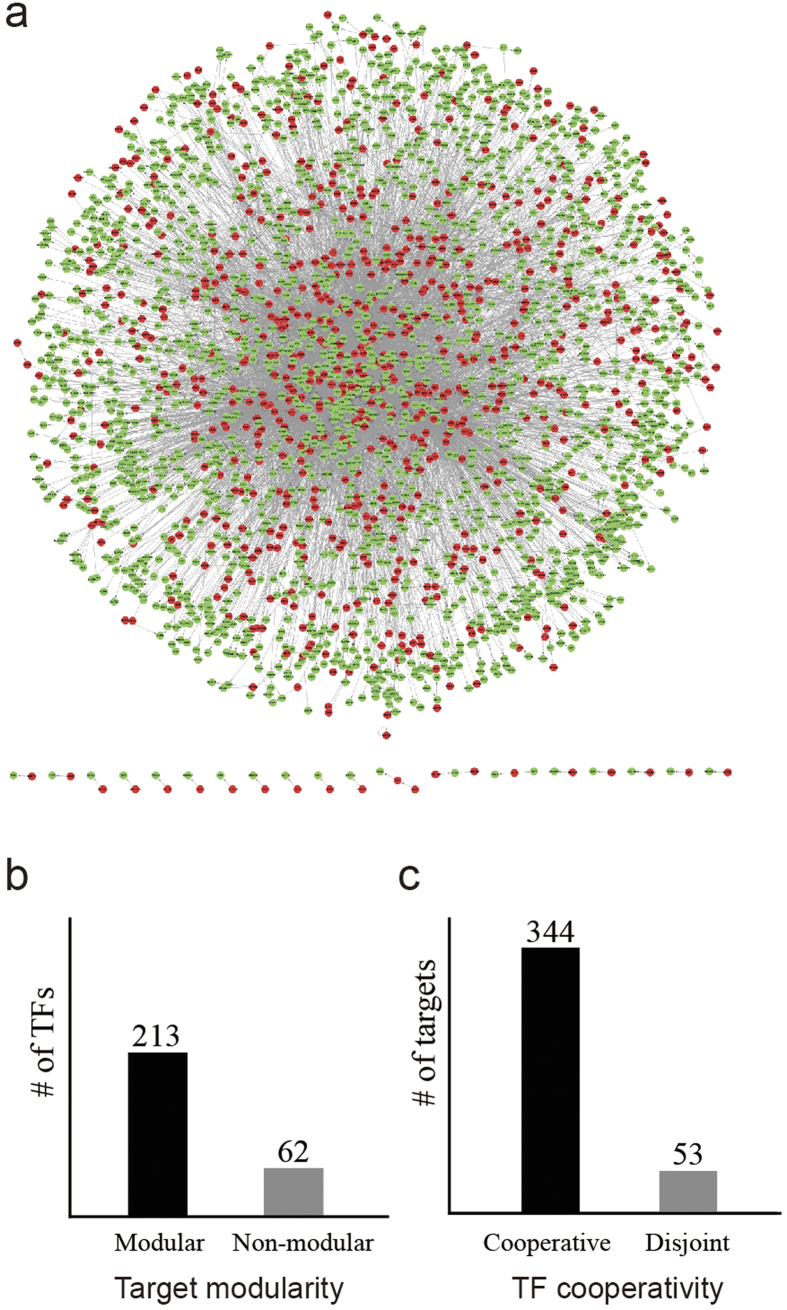
(**a**) A network of TF (red nodes) and non-TF genes (green nodes) based on the regulatory interactions from TRRUST is shown. (**b**) Bar graphs show the number of TFs for two classes based on the different modularity of their targets. Only TFs with more than five target genes were considered for this analysis, resulting in 213 TFs with modular targets and 62 TFs with non-modular targets. (**c**) Bar graphs show the number of target genes for two classes based on the different cooperativity of their TFs. Only target genes regulated by more than five TFs were considered for this analysis, resulting in 344 target genes regulated by cooperative TFs and 53 target genes regulated by disjoint TFs.

The assembly of all identified TF-target interactions in TRRUST reveals a complex TRN of 8,015 links (Fig. 2a). This global network model of transcriptional regulation can be used to address more complex questions than simple queries for interacting molecules. TFs are fundamental regulators of cellular processes, which are generally operated by functionally coherent genes. Thus, target genes regulated by the same TF tend to be modular^13^, often comprising protein complexes or pathways. This target modularity has been used to remove false-positive targets detected from genome-scale ChIP-chip/seq experiments^17^. Given that our database contains only highly reliable TF-target interactions derived from the literature, we expected that a majority of the database TFs would be a highly modular group of target genes. To measure the functional modularity of a group of target genes, we leveraged a genome-wide functional network for humans, HumanNet^18^. If a group of target genes belong to a functional module, then these genes might be well connected in a functional gene network. We measured the significance of an observed ‘within-group edge count’ of the target groups for 275 TFs with no less than five targets by permutation tests using 1,000 groups with the same number of random genes. We classified TFs by target modularity, i.e., TFs with modular targets and TFs with non-modular targets, using a stringent significance threshold (*P* < 0.01). We found that ~75% of the tested TFs (i.e., 213 of 275 TFs) have modular targets (Fig. 2b), which indicates a high level of target modularity among human TFs in the TRRUST database.

A single target gene also can be regulated by synergistic interactions between multiple TFs^14^. This cooperative regulation often is mediated by direct physical interactions among TFs. Therefore, we can test and visualize cooperativity among TFs for a target gene using TF-TF physical interaction data. We measured the cooperativity of a group of TFs that regulate the same target gene by employing literature-curated protein-protein interactions derived from major databases^19^,^20^,^21^,^22^,^23^,^24^ and similar approaches as for the analysis of target modularity. For this analysis, we also used only target genes regulated by no less than five TFs. Similar to the target modularity measurement, the significance of the observed ‘between-group edge count’ for each group of TFs for a target gene was measured by permutation tests using 1,000 groups with the same number of random genes. Similarly, we classified target genes by TF cooperativity, i.e., targets regulated by cooperative TFs and targets regulated by disjoint TFs, using a stringent significance threshold (*P* < 0.01). We found that ~87% of the targets (344 of 397 targets for analysis) are regulated by cooperative TFs (Fig. 2c), which supports our current view of the transcriptional regulatory architecture.

### An interactive web server for analysing a literature-curated human TRN

**Figure 3:**
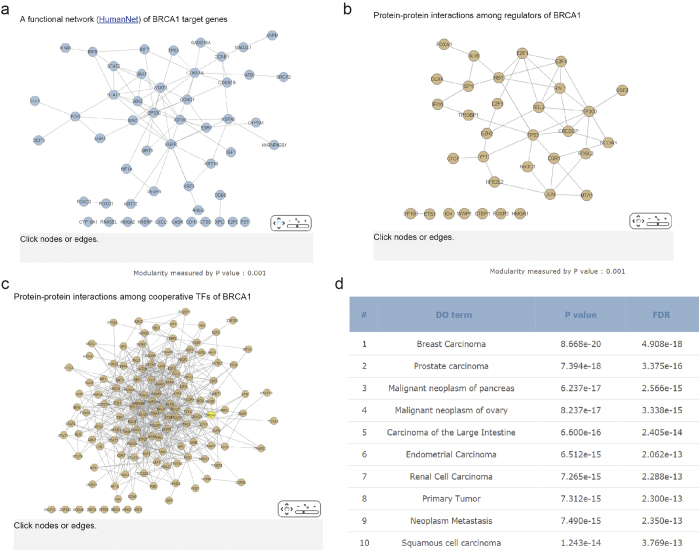
Selective screenshots from TRRUST search results for an example query gene, BRCA1, are shown. (**a**) A functional network of BRCA1 target genes based on HumanNet links is shown. (**b**) The physical interaction network of TFs that regulate BRCA1 based on literature-curated protein-protein interactions derived from major databases is shown. (**c**) A network of TFs that are predicted to cooperate with BRCA1 based on literature-curated protein-protein interactions derived from major databases is shown. (**d**) Disease Ontology terms prioritized for BRCA1 are listed. The top three associated diseases, breast carcinoma, prostate carcinoma, and malignant neoplasm of pancreas, are all validated by the literature.

To perform a database query, users submit a gene name to the search page of the TRRUST web server (http://www.grnpedia.org/trrust), which returns not only the regulatory interactors of the query gene but also other information that facilitates the functional interpretation of human TFs: i) a list of targets, their modularity measure, and functional network (for TF queries only); ii) a list of TF regulators, their cooperativity measure, and the TF-TF physical interaction network (for any query gene); iii) a list of cooperating TFs and a map of the TF-TF physical interactions between them (for TF queries only); and iv) a list of associated pathways and diseases (for TF queries only). The results of an example query using BRCA1 are presented as selective screenshots in Fig. 3.

The TRRUST web server returns a list of BRCA1 targets as well as their functional network from links in HumanNet^18^ (Fig. 3a). High connectivity among the BRCA1 targets suggests that BRCA1 regulates functionally coherent targets. A network of TFs that regulate BRCA1 also is shown by literature-curated protein-protein interactions derived from major databases^19^,^20^,^21^,^22^,^23^,^24^ (Fig. 3b). High connectivity among the TFs suggests that BRCA1 also is regulated by a group of cooperative TFs. The TRRUST web server also infers TFs that might cooperate with a query TF, BRCA1, by measuring the significance of target overlap. TFs that share at least two targets with BRCA1 by high statistical significance [i.e., false discovery rate (FDR) < 0.05, hypergeometric test] are reported along their interaction network (Fig. 3c). The top three cooperative TFs for BRCA1 turned out to be TP53, RELA, and NFKB1. All networks described above are visualized by Cytoscape Web^25^, which is installed on the TRRUST server.

The TRRUST server also prioritizes associated pathways and diseases for a query TF. The significance of associations between a set of target genes regulated by the query TF and a gene set for a pathway or disease was measured by the hypergeometric test across all gene sets with more than five member genes derived from Disease Ontology^26^, KEGG^27^, or Gene Ontology biological process^28^. The server returns all disease/pathway terms associated by FDR < 0.05. For BRCA1, we identified ‘breast carcinoma’, ‘prostate carcinoma’, and ‘malignant neoplasm of pancreas’ as top candidate diseases (Fig. 3d), which were all validated by the literature^29^,^30^,^31^.

Users can freely download the edge information for the TF-target regulatory interactions of TRRUST in both TSV (tab-separated values) and BioC^32^ formats from the download page.

### TRRUST as a benchmark for human TRNs

**Figure 4:**
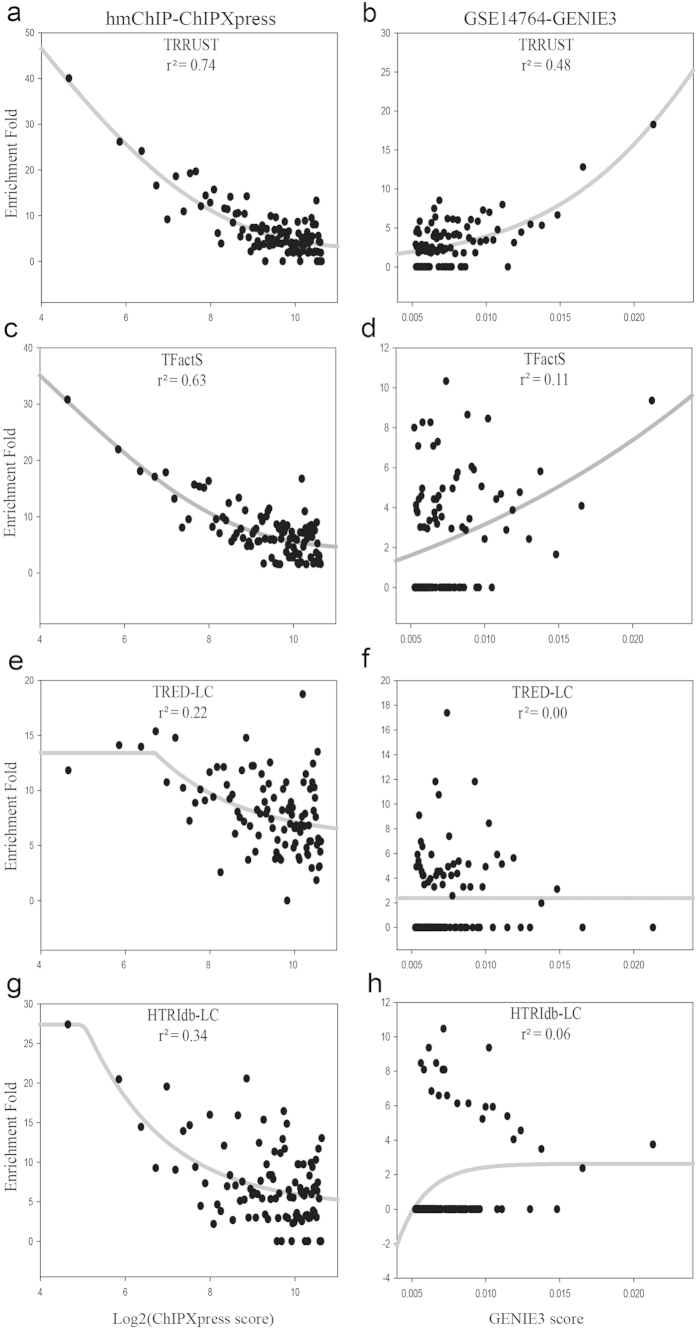
Scatter plots representing the relationship between scores from algorithms (*x*-axis) and the enrichment fold for TRRUST (**a**,**b**), TFactS (**c**,**d**), TRED-LC (**e**,**f**) and HTRIdb-LC (**g**,**h**) gene pairs (*y*-axis) for inferred human TRNs are shown. TF-target interactions inferred from ChIP-chip/seq data of hmChIP database were scored by the ChIPXpress algorithm (**a**) and those from a series of microarray samples from the Gene Expression Omnibus database (GSE14764) were scored by the GENIE3 algorithm (**b**). The enrichment fold was measured for each of successive bins of 1,000 links, which were sorted by algorithm scores. We found best regressions between algorithm scores and the enrichment of benchmarking TF-target interactions using a sigmoidal curve fit for all tested databases. TRRUST exhibits substantially better correlation for the hmChIP-ChIPXpress (Fig. 4a, *r*^*2*^ = 0.74) and GSE14764-GENIE3 (Fig. 4b, *r*^*2*^ = 0.48) TRNs than the other databases (Fig. 4c–h). We used the most significant 100,000 TF-target interactions for all benchmarking analyses, and computed the logarithm of the original ChIPEXpress score due to the highly biased score distribution for the low score range.

Our main motivation for the development of the TRRUST database was to establish a reference database of TF-target interactions for benchmarking reconstructed human TRNs. To test the benchmarking power of the TRRUST data, we used two inferred human TRNs: i) a published TRN inferred from a combined data set of ChIP-chip/seq from the hmChIP database^33^ and various related gene expression data using the ChIPXpress^34^ algorithm and; ii) an unpublished TRN inferred from a series of microarray samples from Gene Expression Omnibus (GEO)^35^ and GSE14764^36^ using the GENIE3^37^ algorithm. The benchmarking power of a given set of TF-target interactions was assessed by their enrichment for each of successive bins of 1,000 inferred regulatory interactions, which were sorted by algorithm scores. To compare TRRUST with other databases of literature-curated TF-target interactions in benchmarking human TRNs, we performed the same assessment for TFactS, TRED-LC, and HTRIdb-LC. As illustrated in Fig. 4, the enrichment of database TF-target interactions is highest for the bin of top-scored interactions, and gradually declines as score decreases in both inferred TRNs. A sigmoidal curve generally shows the best fit for the tested data. We observed the best correlation between algorithm scores and benchmarking interactions enriched by TRRUST in both TRNs (Fig. 4a,b). In contrast, the other databases exhibited relatively weaker correlations for the same TRNs (Fig. 4c–h). These results suggest that TRRUST provides a reliable benchmark for computationally inferred human TRNs from high-throughput data.

## Discussion

A set of gold-standard TF-target interactions to benchmark inferred regulatory interactions from high-throughput data is a vital data tool for the reconstruction of genome-scale human TRNs. In this paper, we presented the largest publicly available database for literature-curated regulatory interactions to date, TRRUST. This new database of human TF-target interactions has several useful features, including the annotations for mode-of-regulation, the incorporation of functional and physical interaction networks for testing target modularity and TF cooperativity, the identification of cooperating TFs, and the prioritization of associated pathways or diseases to a TF. The context information about TFs and targets will facilitate the functional interpretation of given TFs and their target genes in the regulatory networks. The information about mode-of-regulation also will be useful for mechanistic studies of transcriptional regulation. Most importantly, we demonstrated that TF-target interactions of TRRUST can benchmark inferred TRNs from the computational analysis of high-throughput data. Taken together, we conclude that TRRUST will be a useful reference database of TF-target regulatory interactions for the study of TF functions and the reverse engineering of human transcriptional regulatory programs.

## Methods

### Sentence-based text mining

We first filtered ~20 million abstracts from the Medline2014 database using the MeSH descriptor ‘Humans’ to consider only human biology studies. This filter returned 7,740,272 abstracts. Human biology studies also could be found among articles without the ‘Humans’ MeSH term; therefore, this filtering may generate false negatives. However, we empirically found that this filtering step greatly reduces false positives, which is critical to achieving a high retrieval rate during manual curation. For example, many mouse and zebrafish gene names are identical to human gene names. From the 7,740,272 abstracts for human biology, we extracted 57,360 candidate sentences, which needed to contain at least one TF name and another gene name based on our definition. The list of TF genes was derived from Ravasi *et al.*^15^, which is based on the manual curation of several sources: i) the TRANSFAC database; ii) genes annotated by the Gene Ontology (GO) term ‘transcription factor’; iii) genes that contain the word ‘transcription’ in the Entrez description field; and iv) manually curated TF genes by Roach *et al.*^16^ After further curation, this list included 1,984 TFs.

The candidate sentences were subjected to our two-step text-mining procedure. The first step established gold-standard sets of positive and negative candidate sentences for TF-target regulatory interactions. To increase the number of positive sentences, we began our manual curation with sentences that contained commonly used words in the study of transcriptional regulation, such as ‘regulate’, ‘control’, ‘bind’, ‘activate’, ‘enhance’, ‘induce’, ‘repress’, ‘inhibit’, ‘transcription factor’, ‘expression’, ‘promoter’, ‘mRNA’, and ‘target’. A total of 17,409 candidate sentences were subjected to manual curation, and 4,524 and 12,885 sentences were assigned to the gold-standard positive and negative sets, respectively. These gold-standard sentences then were used to prioritize the remaining candidate sentences for the next round of manual curation.

In the second step, we prioritized the remaining candidate sentences based on the frequency difference of each word between the gold-standard positives and negatives. We devised the following score *S* for a given sentence composed of a series of *n* words:


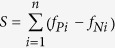


where *f*_*Pi*_ is the frequency of the *i*^th^ word in gold-standard positive sentences and *f*_*Ni*_ is the frequency of the *i*^th^ word in gold-standard negative sentences. All the remaining candidate sentences were ranked by *S*, and the top 6,000 sentences were subjected to the next round of manual curation. The 6,000 curated sentences also can be used to update the set of gold-standard candidate sentences to re-prioritize the remaining and incoming sentences to further improve the retrieval rate in future manual curations. For the current version of TRRUST, a total of 23,409 sentences were subjected to two rounds of manual curation, and 8,015 unique TF-target regulatory interactions were identified.

### Manual curation

We conducted manual curation to identify TF-target regulatory interactions based on the following criteria. For a candidate sentence, we confirmed that official gene symbols appeared in the sentence. For the sentences that passed the gene name criterion, we identified a TF-target regulatory interaction from a sentence: (i) if the sentence indicated that the TF acts as a transcriptional regulator of the target gene; (ii) if a change in the activity of the TF influences the expression of the target gene; (iii) if the TF binds to the promoter region of the target gene; or (iv) if a mutation of the TF binding site on the promoter of the target gene influences the expression of the gene. All primarily identified TF-target regulatory interactions were confirmed by a more experienced curator.

During the manual curation of the 23,409 extracted sentences, many sentences were excluded for several reasons. First, many gene names have aliases that often overlap with non-gene names, such as cell names (e.g., T), chemical names (e.g., SIM), antigen names (e.g., LPS), and regulatory element names (e.g., CRE). Second, some mouse and rat genes have names identical to those of humans. For this reason, we checked whether the gene names in a sentence were human by reading its associated abstract. Third, sentences were excluded if the relationship between the cited genes did not involve transcriptional regulation. For example, many sentences discussed the regulation of protein activity. Additional ambiguities during the curation of sentences were resolved by carefully examining their associated abstracts. We finally identified 8,015 interactions from 6,851 sentences extracted from 6,175 abstracts.

### Extracting information about mode-of-regulation

If a sentence suggested a mode-of-regulation, i.e., either activation or repression, for the given TF-target regulatory interaction, we collected and deposited the information along with the given TF-target interaction in the database. An example sentence that describes activation is “Moreover, a co-expression of p300 and ATF-2 enhanced the promoter activity of IFN gamma gene.” An example sentence that describes repression is “ESE-1 regulates MMP-9 expression in a negative manner and the ets binding site on the MMP-9 promoter contributed to suppression by ESE-1.” If there was no indication about the mode-of-regulation in either the given sentence or its associated abstract, then we assigned the term ‘unknown’ for the mode-of-regulation of the TF-target regulatory interaction. In addition, if multiple sentences from either a single or multiple abstracts indicated both activation and repression, we listed the TF-target interactions twice with each mode-of-regulation along with the supporting Medline abstract for each mode.

### TF-target interactions in other public databases

We downloaded the regulatory interactions from TFactS^9^, HTRIdb^11^, and ORegAnno^12^ as batch files. For TFactS, we used TF-target interactions for human only. For ORegAnno, TF-target interactions by ChIP-seq experiments were excluded. The regulatory interactions from TRED^10^ were manually collected by querying all searchable TFs, because TRED does not offer downloadable batch files. We filtered the interactions from TRED for targets with binding quality ‘known’ to retrieve only literature-curated interactions of the database (TRED-LC). HTRIdb also contains many regulatory interactions derived from high-throughput DNA-binding experiments, which need to be excluded from the literature-curated HTRIdb (HTRIdb-LC). We excluded interactions from HTRIdb that were labelled with the technique term ‘Chromatin Immunoprecipitation coupled with microarray’ or ‘Chromatin Immunoprecipitation coupled with deep sequencing’.

### Measuring target modularity and TF cooperativity

To measure the functional modularity of the target genes for each TF, we applied a functional gene network, HumanNet^18^, on the TRRUST web server. By assuming that a modular group of genes has more within-group links than groups of random genes, we assessed modularity via the significance of the observed ‘within-group edge count’ among genes targeted by the same TF. The significance test was based on a null model by permutation tests with 1,000 groups of random genes for the same group size. For the target modularity test, we considered only TFs with no less than five targets. We classified TFs by target modularity, i.e., TFs with modular targets and TFs with non-modular targets, using a stringent significance threshold (*P* < 0.01). We also assessed TF cooperativity using a similar approach on a set of 10,119 literature-curated protein-protein interactions between TFs derived from major databases^19^,^20^,^21^,^22^,^23^,^24^. The same significance threshold was used to divide the target genes into two classes: targets regulated by cooperative TFs and targets regulated by disjoint TFs.

### Enrichment of TRRUST TF-target interactions in human TRNs

The hmChIP database^33^ provides 148 files of TF-target links for 69 TFs. Each file contains a ranked list of target genes for a TF and scores calculated by the ChIPXpress algorithm^34^ based on ChIP-chip/seq data and various gene expression data. We combined those files to construct one example of a human TRN that comprises TF-target relationships ranked by score. We applied the GENIE3 algorithm^37^ to GSE14764^36^ microarray data to infer another example of a human TRN. More significant regulatory interactions have lower scores by the ChIPXpress algorithm and higher scores by the GENIE3 algorithm. We measured the enrichment of database TF-target interactions for each bin of 1,000 inferred interactions in the two example TRNs.

## Additional Information

**How to cite this article**: Han, H. *et al.* TRRUST: a reference database of human transcriptional regulatory interactions. *Sci. Rep.* **5**, 11432; doi: 10.1038/srep11432 (2015).

## Figures and Tables

**Table 1:** A summary of TRRUST and four other databases for literature-curated TF-target regulatory interactions in human.

**Database**	**# of TFs**	**# of non-TF genes**	**# of Links**
TRRUST	748	1,975	8,015
TFactS	277	1,932	4,311
TRED-LC*	119	1,582	3,332
HTRIdb-LC*	282	1,358	2,284
ORegAnno	67	122	202

^*^Only literature-curated (LC) interactions in the database were considered for this study.
